# A Screening Method for the Isolation of Polyhydroxyalkanoate-Producing Purple Non-sulfur Photosynthetic Bacteria from Natural Seawater

**DOI:** 10.3389/fmicb.2016.01509

**Published:** 2016-09-21

**Authors:** Mieko Higuchi-Takeuchi, Kumiko Morisaki, Keiji Numata

**Affiliations:** Enzyme Research Team, Biomass Engineering Research Division, RIKEN Center for Sustainable Resource ScienceWako, Japan

**Keywords:** polyhydroxyalkanoates, marine purple non-sulfur photosynthetic bacteria, seawater, nutrient-rich conditions, 16S rRNA

## Abstract

Polyhydroxyalkanoates (PHAs) are a family of biopolyesters accumulated by a variety of microorganisms as carbon and energy storage under starvation conditions. We focused on marine purple non-sulfur photosynthetic bacteria as host microorganisms for PHA production and developed a method for their isolation from natural seawater. To identify novel PHA-producing marine purple non-sulfur photosynthetic bacteria, natural seawaters were cultured in nutrient-rich medium for purple non-sulfur photosynthetic bacteria, and twelve pink- or red-pigmented colonies were picked up. Gas chromatography mass spectrometry analysis revealed that four isolates synthesized PHA at levels ranging from 0.5 to 24.4 wt% of cell dry weight. The 16S ribosomal RNA sequence analysis revealed that one isolate (HM2) showed 100% identity to marine purple non-sulfur photosynthetic bacteria. In conclusion, we have demonstrated in this study that PHA-producing marine purple non-sulfur photosynthetic bacteria can be isolated from natural seawater under nutrient-rich conditions.

## Introduction

Polyhydroxyalkanoates (PHAs) are biopolyesters that many microorganisms accumulate as intracellular reservoirs of carbon and energy. PHAs have attracted increasing attention due to their biomass-based origin and biodegradability (Numata et al., 2009). However, PHA production requires a costly carbon source, such as sugars or plant oils. The use of photosynthetic organisms to produce materials is clean and eco-friendly because the energy for growth is derived from the sun, and the carbon is derived from carbon dioxide in the air. Although efforts have been made to utilize photosynthetic organisms such as cyanobacteria and higher plants for PHA production, high PHA production has not been achieved to date (Osanai et al., 2013, 2014). Anoxygenic photosynthetic bacteria are known to produce more PHA (Liebergesell et al., 1991) than cyanobacteria and higher plants. However, PHA production by photosynthetic bacteria has been studied in a small number of freshwater purple non-sulfur photosynthetic bacteria strains, such as *Rhodospirillum rubrum* (Brandl et al., 1989; Clemente et al., 2000), *Rhodopseudomonas sphaeroides* (Lorrungruang et al., 2006) and *Rhodobacter capsulatus* (Kranz et al., 1997). Purple non-sulfur photosynthetic bacteria have several advantages over other photosynthetic bacteria. One advantage is that purple non-sulfur photosynthetic bacteria can grow either aerobically in the dark or anaerobically in the light. In addition, purple non-sulfur photosynthetic bacteria can utilize various electron acceptors, i.e., they are facultative photosynthetic bacteria (Mcewan, 1994; Basak and Das, 2007). To take advantage of these properties, purple non-sulfur photosynthetic bacteria have been tested for use in a variety of applications, including not only PHA production but also the purification of industrial wastewater (Kim et al., 2004; Wu et al., 2012) and hydrogen production (Basak and Das, 2007).

Marine microorganisms are important bioresources and expected to produce new value-added compounds, including PHA (Numata and Doi, 2012; Numata et al., 2013; Numata and Morisaki, 2015). Cultivation under marine conditions offers several advantages for the industrial production of PHA. For example, high concentrations of salts inhibit the growth of salt-sensitive bacteria species. In addition, filtered sterilized seawater can be used as a culture medium. However, PHA production under marine conditions has been reported using certain types of marine bacteria (Lopez et al., 2009; Shrivastav et al., 2010; Numata and Doi, 2012). Although PHA production by marine purple non-sulfur photosynthetic bacteria has been reported by a few groups (Chowdhury et al., 1996; Xiao and Jiao, 2011), the details of the PHA synthesis were not studied thoroughly. The small number of studies on PHA synthesis by marine purple non-sulfur photosynthetic bacteria, even though they are important host bacteria to produce PHA, is because no isolation method of PHA-producing marine purple non-sulfur photosynthetic bacteria has been established until now.

Many screening methods have been developed to detect microorganisms that accumulate PHAs. The methods most widely used for detecting PHAs are staining techniques using Nile red (Spiekermann et al., 1999), Nile blue A (Ostle and Holt, 1982) and Sudan Black (Steinbuchel et al., 1987). Using these staining techniques, a variety of PHA-producing bacteria and mutants have been isolated. However, this method is unable to discriminate PHA and lipids. Additionally, it is necessary to provide nutrient limitation conditions and suitable carbon sources to the bacterial cells to induce PHA production. Purple photosynthetic bacteria contain bacteriochlorophyll *a* or *b* and various types of carotenoids. Staining methods are not suitable for purple non-sulfur photosynthetic bacteria because these pigments interfere with staining and detection. The other method for identifying PHA-producing bacteria is the polymerase chain reaction (PCR) amplification of PHA synthesis genes using degenerate primers (Sheu et al., 2000; Shamala et al., 2003). This technique is a rapid and accurate detection system for screening large numbers of environmental isolates. However, this technique leads to detection errors because of the non-specific PCR amplification and lack of PCR products due to degenerate primer sequences. Furthermore, this method cannot detect PHA itself, and hence PHA and its induction conditions must be determined after the discovery of PHA biosynthesis genes. Therefore, it is necessary to develop a screening method for the isolation of PHA-producing marine purple non-sulfur photosynthetic bacteria.

In a previous study, we evaluated the potential use of marine purple photosynthetic bacteria for PHA production (Higuchi-Takeuchi et al., 2016). Our study demonstrated that marine purple photosynthetic bacteria were good host microorganisms for industrial PHA production using marine resources. The aim of our study is to develop an approach to isolate PHA-producing purple photosynthetic bacteria from natural marine environments. We found that purple sulfur photosynthetic bacteria did not accumulate PHA under nutrient-rich conditions, whereas some species of purple non-sulfur photosynthetic bacteria did accumulate PHA without nutrient limitation, in contrast to the well-known PHA-producing soil bacteria (Higuchi-Takeuchi et al., 2016). Purple photosynthetic bacterial cultures can be purple, red, brown or orange because of the various types of carotenoids and bacteriochlorophyll. Based on these observations, in this study, we isolated pigmented bacteria under nutrient-rich conditions as PHA-producing purple non-sulfur photosynthetic bacteria from natural seawaters.

## Materials and Methods

### Culture Conditions and Seawater Sampling

Purple non-sulfur bacteria were grown in culture medium (JCM medium number 520)^1^. The composition was modified based on the medium used for isolation of purple non-sulfur bacteria (Biebl and Pfennig, 1981). Medium was composed of the following components per liter: KH_2_PO_4_ (0.5 g); CaCl_2_⋅2H_2_O (0.25 g); MgSO_4_ ⋅7H_2_O (3.0 g); NH_4_Cl (0.68 g); NaCl (20 g); sodium malate (3.0 g); sodium pyruvate (3.0 g); yeast extract (0.4 g); ferric citrate (5 mg); vitamin B_12_ (2 mg); ZnCl_2_⋅5H_2_O (70 μg); MnCl_2_⋅4H_2_O (100 μg); H_3_BO_3_ (60 μg); CoCl_2_⋅6H_2_O (200 μg); CuCl_2_⋅2H_2_O (20 μg); NiCl_2_⋅6H_2_O (20 μg) and Na_2_MoO_4_⋅H_2_O (40 μg). The pH was adjusted to 6.8. Yeast extract (Culture media grade) and ferric citrate (BioReagent grade) were purchased from Becton Dickinson (Franklin Lakes, NJ, USA) and Sigma-Aldrich (St. Louis, MO, USA), respectively. The rest chemicals (JIS Special Grade) were purchased from Wako Chemicals (Osaka, Japan).

For screening, aliquots of 100 μL of each seawater sample from all sampling points were spread in agar plates. Seawaters were cultured multiple times until pigmented-colony was obtained. Maximum number of replicates was six. Six replicates were carried out using seawaters from Naha, Tokyo-Bay and Sakata. The number of replicates was five in Omura-Bay sample, six in Tokyo-Bay, four in Aioi-Bay, two in Takamatsu and two in Yokohama. Seawaters were cultured aerobically under continuous far-red LED light conditions (730 nm, 8 Wm^-2^) at 30°C. Addition of larger amount of seawater and concentration of seawater using filter paper might be effective for obtaining the large number of bacterial colonies. The isolated colonies were streaked onto other plates for purification. Streak plating should be done until the pure colonies from single bacteria are isolated. Transferring of small volume of cell culture (1.5 mL) into large scale culture (50 mL) is recommended for better bacterial growth. The isolates were transferred to 1.5 mL capped plastic tubes filled with liquid medium and cultured for 7–10 days under continuous far-red LED light conditions at 30°C and then transferred in 50 mL screw capped plastic tubes filled with liquid medium and cultured without stirring for 10 days. To transfer into liquid culture, transferring a small amount of bacteria cells (not a single colony) using inoculating loops under aseptic conditions is preferred for good bacterial cell growth. Bacterial cells were stored as 10% glycerol (JIS Special Grade, Wako Chemicals, Osaka, Japan) stocks at -80°C for further experiments.

About 50 mL of natural seawaters were collected from Naha (Okinawa), Omura-Bay (Nagasaki), Tokyo-Bay (Tokyo), Aioi-Bay (Hyogo), Sakata (Yamagata), Takamatsu (Kagawa) and Yokohama (Kanagawa) in Japan. Sampling points were randomly selected. One seawater sample was collected from the sea surface in each point. Sampled seawaters were stored at 4°C in the dark until use.

### Analysis of PHA Content and Composition

The PHA content characterization method was modified slightly from a previous study (Chuah et al., 2013). Approximately 0.5–2 mg lyophilized cells were incubated in 1 mL of 100% ethanol at 70°C for 1 h to remove pigments. The cells were then subjected to ethanolysis in the presence of 250 μL chloroform (JIS Special Grade, Wako Chemicals, Osaka, Japan), 100 μL hydrochloric acid (JIS Special Grade, Wako Chemicals, Osaka, Japan) and 850 μL ethanol (JIS Special Grade, Wako Chemicals, Osaka, Japan) at 100°C for 4 h. After cooling, 1 mL of phosphate buffer (pH 8.1) was added to the reaction mixture and then neutralized with 0.65 N NaOH. After centrifugation at 1,500 rpm for 5 min (CF16RN, Hitachi-Koki, Tokyo, Japan), the lower chloroform layer was filtered through anhydrous sodium sulfate (JIS Special Grade, Wako Chemicals, Osaka, Japan) and incubated with molecular sieves 4A (Nacalai tesque, Kyoto, Japan) for 30 min. The PHA content and composition were determined using a gas chromatography–mass spectrometry (GC-MS) apparatus (GCMS-QP2010 Ultra, Shimadzu, Tokyo, Japan) equipped with a 30 mm × 0.25 mm DB-1 capillary gas chromatography column (Agilent Technologies, Santa Clara, CA, USA). For analysis, 1 μL of sample solution was injected with helium as a carrier gas (3.30 mL min^-1^). The following temperature program was used to separate ethyl esters: 45°C for 1 min, temperature ramp of 7°C per min to 117°C. The interface and ion source temperatures were 250°C and 230°C, respectively. The 3HB content was determined using a calibration curve.

### Extraction of PHAs and ^1^H NMR analysis

PHA extraction and purification was carried out according to the method reported in (Brandl et al., 1989). PHAs were extracted from 428 mg lyophilized cells using about 100 mL of chloroform (JIS Special Grade, Wako Chemicals, Osaka, Japan). The chloroform extracts were filtered and concentrated using a rotary vacuum evaporator (NA-1, AS ONE, Osaka, Japan), and the chloroform-extracted PHAs were purified by precipitation with hexane (JIS Special Grade, Wako Chemicals, Osaka, Japan) of more than 10 times the volume of the solvent. The precipitate was filtered and then air-dried without vacuum as a convenient way overnight at room temperature. The polymer solution was concentrated again using a rotary vacuum evaporator and purified by precipitation with cold methanol (JIS Special Grade, Wako Chemicals, Osaka, Japan) of more than 10 times the volume of the solvent. The purified PHA precipitate was air-dried overnight at room temperature.

The purified PHAs were analyzed by proton nuclear magnetic resonance (^1^H NMR; JNM-Excalibur 270; JEOL, Ltd., Tokyo, Japan) to determine their chemical structures and compositions. The measuring frequency was 499.87 MHz. The sample for NMR analysis was dissolved at a concentration of 4 mg/mL in CDCl_3_ with 0.05% (v/v) tetramethylsilane (TMS; Wako Pure Chemical Industries Ltd., Osaka, Japan).

### Determination of 16S Ribosomal RNA Sequences and Phylogenetic Analysis

DNA was extracted using the DNeasy Blood & Tissue Kit (Qiagen, Hilden, Germany). The 16S ribosomal RNA sequences were determined using the Bacterial 16S rDNA PCR kit according to the manufacturer’s protocol (Takara Bio, Shiga, Japan).

The phylogenetic tree was prepared based on the 16S ribosomal RNA sequences of the 13 selected purple photosynthetic bacteria and constructed using Phylogeny^2^ (Castresana, 2000; Edgar, 2004; Anisimova and Gascuel, 2006; Dereeper et al., 2008; Guindon et al., 2010). NJplot was used to display the phylogenetic tree (Perriere and Gouy, 1996).

## Results and Discussion

**FIGURE 1:**
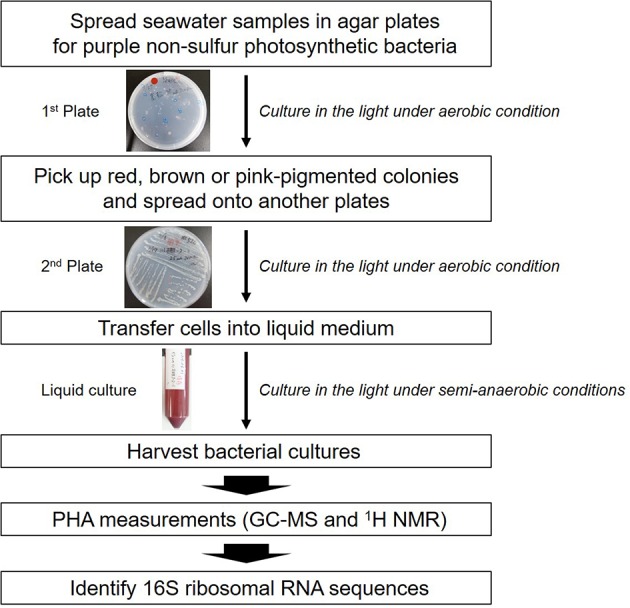
**Isolation scheme for PHA-producing purple non-sulfur photosynthetic bacteria**.

The screening strategy for PHA-producing purple non-sulfur photosynthetic bacteria is shown in **Figure 1**. In the case of well-known PHA-producing bacteria, PHA production is induced under nutrient(s)-free conditions such as nitrogen, phosphorus or sulfur. We previously found that purple non-sulfur photosynthetic bacteria could accumulate PHA without nutrient deficiency (Higuchi-Takeuchi et al., 2016). Nutrient deficiency conditions are not appropriate for the isolation of PHA-producing purple non-sulfur photosynthetic bacteria from natural environments because such conditions result in poor bacterial growth. Therefore, seawaters were cultured in nutrient-rich agar plates for the growth of marine purple non-sulfur photosynthetic bacteria. To select PHA-producing purple non-sulfur photosynthetic bacteria by color, the plates were cultivated under continuous far-red light conditions. After extended cultivation, a red- or pink-pigmented colony typical of purple photosynthetic bacteria was picked up. The selected colony was cultured in liquid medium, and the harvested cells were subjected to GC-MS analysis to measure their PHA content. Lastly, the 16S rRNA sequences were determined to identify the bacterial species.

To obtain purple non-sulfur photosynthetic bacteria colonies, each seawater sample from all sampling points was spread in nutrient-rich agar plates for the growth of purple non-sulfur photosynthetic bacteria and cultured aerobically in the light. Most bacterial colonies appeared white, cream or yellow in color after 1 day (Supplementary Figure 1C). Some bacteria produced pink or red colonies after 4–7 days (Supplementary Figures 1A,B). Extended incubation times may permit the appearance of more pigmented-colonies although they result in growth of bacteria other than purple non-sulfur photosynthetic bacteria. A total of twelve red or pink colonies were obtained from four seawater samples (**Table 1**). HM1 and HM2 were obtained from the seawater of Omura-Bay; HM3 and HM4 were obtained from the seawater of Takamatsu; HM5 was obtained from the seawater of Aioi-Bay; and seven isolates (HM5 to HM12) were isolated from the seawater of Yokohama. Pigmented colonies were streaked onto other plates for further isolation (Supplementary Figures 1D–G). The twelve isolates were then transferred to liquid medium and cultured in the light (Supplementary Figures 1H–K). Among the twelve isolates, HM2 exhibited a bright red color in liquid culture (Supplementay Figure 1I). The cell cultures of the other eleven isolates were red in color after centrifugation (right side of Supplementary Figures 1H,J,K), although the liquid culture appeared to exhibit a very faint pink color or no color production (left side of Supplementary Figures 1H,J,K). Based on the results of the liquid cell cultures, HM2 was the only positive strain, namely, purple photosynthetic bacteria.

**Table 1:** PHA content (wt%) of isolates.

Isolates	CDW (mg/L)	PHA (wt%)	Source of isolate
HM1	184	1.0	Omura-Bay (Nagasaki)
HM2	46	24.4	Omura-Bay (Nagasaki)
HM3	127	18.0	Takamatsu (Kagawa)
HM4	50	n.d.	Takamatsu (Kagawa)
HM5	49	n.d.	Aioi-Bay (Hyogo)
HM6	99	0.5	Yokohama (Kanagawa)
HM7	105	n.d.	Yokohama (Kanagawa)
HM8	64	n.d.	Yokohama (Kanagawa)
HM9	119	n.d.	Yokohama (Kanagawa)
HM10	102	n.d.	Yokohama (Kanagawa)
HM11	103	n.d.	Yokohama (Kanagawa)
HM12	95	n.d.	Yokohama (Kanagawa)

*n.d., not detected.*

**FIGURE 2:**
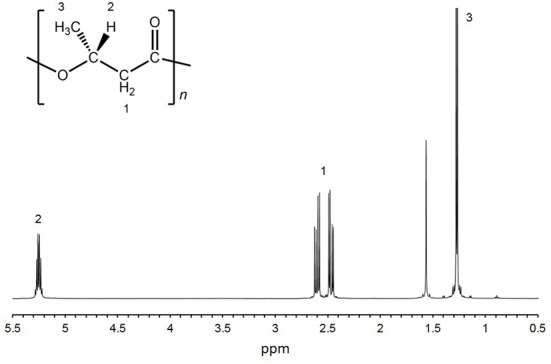
**^1^H-NMR spectra of purified PHA.** H-NMR analysis was conducted using purified PHA from HM2 cells.

Twelve isolates were cultured in growth medium and then measured the cell dry weight (CDW) and PHA content. CDW varied among the isolates, ranging from 46 to 184 mg/L culture (**Table 1**). The PHA content of the twelve isolates was measured by GC-MS. PHA was not detected in eight isolates. Four isolates (HM1, HM2, HM3 and HM6) synthesized PHA at levels ranging from 0.5 to 24.4 wt% of CDW (**Table 1**). HM2 showed the highest PHA accumulation (24.4 wt%). To verify these results, the PHA content and CDW of HM2 were measured using triplicate cultures. The CDW of HM2 was 800 ± 58 mg/L culture, and the PHA content was 24.2 ± 1.8 wt%. GC-MS analysis also revealed that four isolates synthesized a homopolymer of 3-hydroxybutyrate (3HB), namely, poly(3-hydroxybutyrate) (PHB). The chemical structure of the synthesized PHAs was determined by ^1^H NMR with chloroform extracts from HM2 isolate. Methyl, methylene, and methine protons of 3HB units were detected by ^1^H NMR (**Figure 2**). Thus, the ^1^H NMR spectra confirmed the chemical structure of PHB.

**FIGURE 3:**
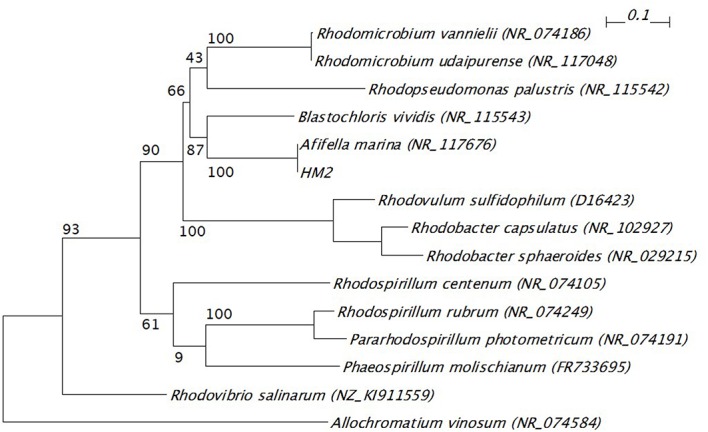
**Phylogenetic tree of 16S ribosomal RNA gene from HM2 and purple non-sulfur photosynthetic bacteria.** The scale bar shows the number of nucleotide substitutions per site. GenBank accession numbers are given in parentheses. The bootstrap values are shown at branch nodes. *Allochromatium vinosum* was used as an outgroup.

The 16S ribosomal RNA sequences of four isolates were determined and compared with the databank contents using Nucleotide BLAST. Two isolates (HM3 and HM6) showed more than 99% sequence identity to *Shewanella basaltis*, and one isolate (HM1) exhibited high similarity to *Marinobacter guineae* (**Table 2**). *S. basaltis* (Chang et al., 2008) and *M. guineae* (Montes et al., 2008) are marine bacteria but not purple non-sulfur photosynthetic bacteria. The isolate HM2, which was positive by color selection in liquid culture and accumulated the largest amount of PHA, showed 100% identity to the purple non-sulfur photosynthetic bacteria *Afifella marina* (Imhoff, 1983) and *Rhodopseudomonas julia* (Kompantseva, 1989) isolated from saline environments, indicating that bright pigmented-color in liquid culture is one of important points for isolation of purple non-sulfur bacteria. A phylogenetic tree was constructed based on the 16S rRNA sequences from HM2 and purple non-sulfur photosynthetic bacteria of the alphaproteobacteria that have been studied by whole-genome analysis (**Figure 3**). HM2 was positioned close to *Blastochloris viridis* in the phylogenetic tree. Further characterization of the isolates will be needed to determine the species.

**Table 2:** 16S ribosomal RNA sequences of isolates.

Isolate	Closest relative	GenBank accession no.	Identity (%)	Identity (match bp/ total bp)
HM 1	*Marinobacter guineae*	KF500390	99.9	1420/1422
HM 2	*Afifella marina*	NR_117676	100.0	1336/1336
	*Rhodopseudomonas julia*	NR_040937		
HM 3	*Shewanella basaltis*	KC534403	99.8	1287/1290
HM 6	*Shewanella basaltis*	KC534403	99.9	1254/1255

The isolation method we described here identified the strain HM2, which showed the highest PHA production among twelve isolates, and the 16S rRNA sequences of this strain were shown to have high similarity to marine purple non-sulfur photosynthetic bacteria. Based on the data on PHA accumulation and CDW, the PHA content of HM2 was calculated to be 199 ± 16 mg/L culture. Brandl et al. (1989) reported production levels of 500 mg/L PHA in *R. sphaeroides* and 390 mg/L PHA in *R. rubrum*. HM2 showed similar levels of PHA production to these strains. Seawater sampling methods were not optimized in this study. Sampling points were randomly selected and only one seawater sample was collected from each sampling point. Further investigations for sampling methods are required to obtain a lot of marine purple non-sulfur photosynthetic bacteria from natural seawaters. Further optimization of factors such as the PHA induction conditions and screening of PHA-producing marine purple non-sulfur photosynthetic bacteria will allow the development of PHA production by photosynthetic organisms.

## Conclusion

One isolate identified in this study accumulated 24.4 wt% PHA, and 16S rRNA gene sequence analysis revealed that this strain showed high similarity to marine purple non-sulfur photosynthetic bacteria. Thus, we successfully developed a screening method to isolate PHA-producing purple non-sulfur photosynthetic bacteria under nutrient-rich and far-red light conditions from the natural environment. With this isolation method, PHA production by marine purple non-sulfur photosynthetic bacteria will be studied widely, leading to green and eco-friendly PHA production from carbon dioxide and marine resources.

## Author Contributions

MH-T and KN conceived and designed the study. KM performed the experiments. MH-T analyzed the data, and MH-T and KN interpreted the data. MH-T drafted the manuscript, and KN and MK approved the manuscript.

## Conflict of Interest Statement

The authors declare that the research was conducted in the absence of any commercial or financial relationships that could be construed as a potential conflict of interest.

## Footnotes

1 http://www.jcm.riken.jp/cgi-bin/jcm/jcm_grmd?GRMD=520

2 http://phylogeny.lirmm.fr/phylo_cgi/index.cgi
