# Supplementary material for: A Screening Method for the Isolation of Polyhydroxyalkanoate-Producing Purple Non-sulfur Photosynthetic Bacteria from Natural Seawater
Source: Front Microbiol. 2016 Sep 21;7:1509. doi: 10.3389/fmicb.2016.01509 (PMC5030216; doi:10.3389/fmicb.2016.01509)
Supplement: Supplementary file 1 [file Image_1.PDF]

Supplementary Material

A screening method for the isolation of polyhydroxyalkanoate-producing purple non-sulfur photosynthetic bacteria from natural seawater

Mieko Higuchi-Takeuchi, Kumiko Morisaki, Keiji Numata*

*** Correspondence:**

Keiji Numata

keiji.numata@riken.jp

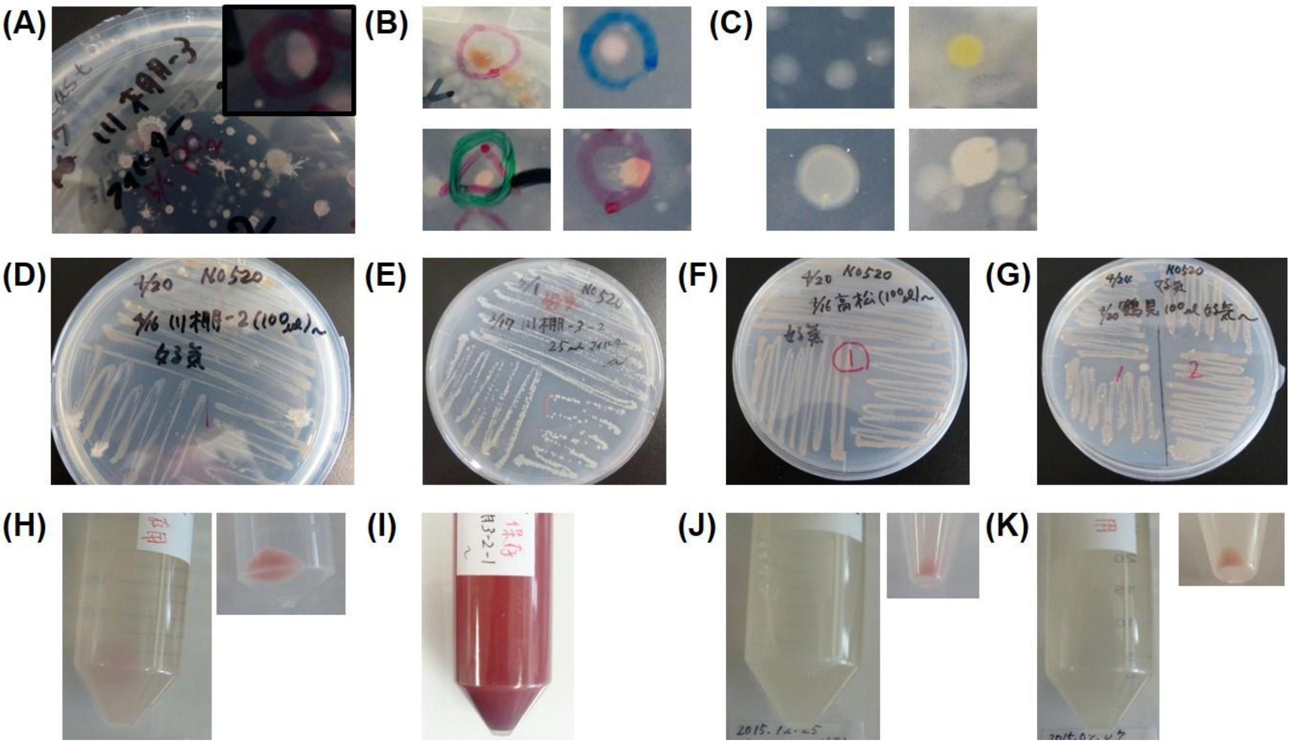

Supplementary Figure 1. Four isolates analyzed in this study. (A) Seawater samples from Omura-Bay were cultured under nutrient-rich conditions. Upper right images show enlarged view of purple non-sulfur photosynthetic bacterial colony. (B) False-positive pigmented colonies of marine bacteria. (C) Negative white, cream or yellow colonies. Pigmented colonies were picked from agar plates and then spread onto other plates (D, E, F and G). Picked cells were cultured in liquid medium (H, I, J and K). Precipitates after centrifugation of the liquid cultures are shown in the right side of liquid cultures. HM1: (A), (D) and (H). HM2: (A), (E) and (I). HM3: (F) and (J). HM4: (G) and (K).
